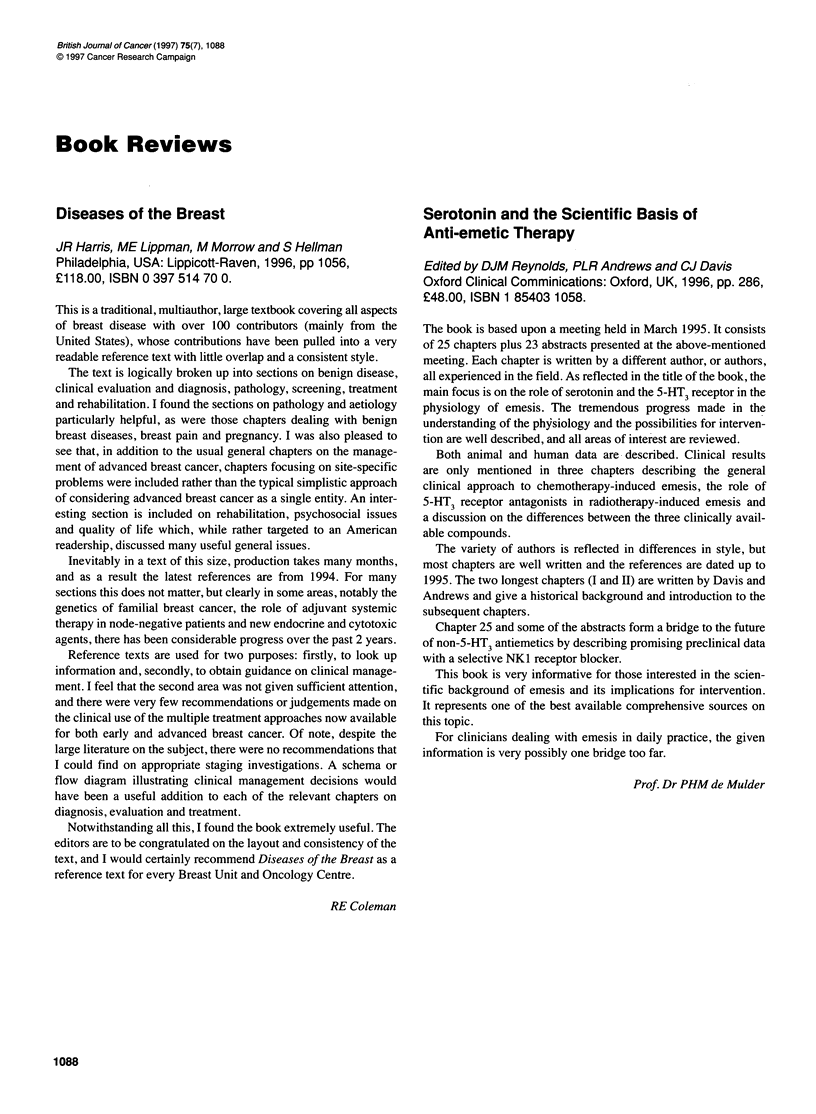# Serotonin and the Scientific Basis of Anti-ematic Therapy

**Published:** 1997

**Authors:** PHM de Mulder


### Serotonin and the Scientific Basis of Anti-emetic Therapy

*Edited by DJM Reynolds, PLR Andrews and CJ Davis*
Oxford Clinical Comminications: Oxford, UK, 1996, pp. 286, £48.00, ISBN 1 85403 1058.

The book is based upon a meeting held in March 1995. It consists of 25 chapters plus 23 abstracts presented at the above-mentioned meeting. Each chapter is written by a different author, or authors, all experienced in the field. As reflected in the title of the book, the main focus is on the role of serotonin and the 5-$HT_3$ receptor in the physiology of emesis. The tremendous progress made in the understanding of the physiology and the possibilities for intervention are well described, and all areas of interest are reviewed.

Both animal and human data are described. Clinical results are only mentioned in three chapters describing the general clinical approach to chemotherapy-induced emesis, the role of 5-$HT_3$ receptor antagonists in radiotherapy-induced emesis and a discussion on the differences between the three clinically available compounds.

The variety of authors is reflected in differences in style, but most chapters are well written and the references are dated up to 1995. The two longest chapters (I and II) are written by Davis and Andrews and give a historical background and introduction to the subsequent chapters.

Chapter 25 and some of the abstracts form a bridge to the future of non-5-$HT_3$ antiemetics by describing promising preclinical data with a selective NK1 receptor blocker.

This book is very informative for those interested in the scientific background of emesis and its implications for intervention. It represents one of the best available comprehensive sources on this topic.

For clinicians dealing with emesis in daily practice, the given information is very possibly one bridge too far.

*Prof. Dr PHM de Mulder*